# Heat Treatment and Storage of Frass From Black Soldier Fly Larvae and Yellow Mealworm Production: Compliance With EU Regulation on Microbiological Quality and Safety

**DOI:** 10.1002/mbo3.70020

**Published:** 2025-05-29

**Authors:** Ann De Volder, Jeroen De Smet, Lotte Frooninckx, David Deruytter, Johan Ceusters, Dries Vandeweyer

**Affiliations:** ^1^ Department of Microbial and Molecular Systems (M^2^S) KU Leuven, Geel Campus, Research Group for Insect Production and Processing (IP&P) Geel Belgium; ^2^ Department of Biosystems, Research Group for Sustainable Crop Production and Protection (SusCroPP) KU Leuven, Geel Campus Geel Belgium; ^3^ Centre of Expertise Sustainable Biomass and Chemistry Thomas More University of Applied Sciences Geel Belgium; ^4^ Insect Research Center, Inagro Rumbeke‐Beitem Belgium

**Keywords:** *E. coli*, Enterococaceae, *Hermetia illucens*, insect rearing residue, *Salmonella*, *Tenebrio molitor*

## Abstract

Insect farming generates substantial amounts of frass, a nutrient‐rich by‐product with valorization potential as fertilizer/soil improver. Marketing is restricted by EU regulations, imposing a reference heat treatment for 1 h at 70°C to reduce the presence of potential pathogens. This study evaluated the impact of heat treatments (50 → 80°C for 15 → 90 min) on microbiological quality and safety of black soldier fly larvae (BSFL) and yellow mealworm (YM) frass, as well as microbial dynamics during storage, before and after reference treatment. Fresh frass showed high microbial counts, but *Salmonella* was absent. Untreated BSFL frass did not meet the EU criteria to allow use as fertilizer, while some YM frass samples did. Reference heat‐treated BSFL and YM frass complied with the EU criteria. *Escherichia coli* counts were below the 1000 cfu/g limit, and *Salmonella* (even when inoculated before treatment) was absent. Only in BSFL frass, Enterococcaceae counts were sufficiently reduced. Milder treatments (temperatures < 70°C and/or times < 1 h) of BSFL frass induced similar reductions of *E. coli* and (inoculated) *Salmonella* but Enterococcaceae counts remained above 1000 cfu/g. In some YM frass samples (inoculated) *Salmonella* was still detected after milder treatment. Refrigerated (4°C) or ambient temperature (26°C–28°C) storage up to 2 weeks of fresh and heat‐treated frass did not increase bacterial counts. To ensure frass microbiological safety whilst preserving quality and reducing costs, tailored treatments seem appropriate. This may be no, milder, or more severe treatment, depending on the microbial load (counts and species type).

## Graphical abstract

Microbiological quality/safety of frass from black soldier fly larvae and yellow mealworm production, before/after heat treatment and/or storage is determined by insect species, frass origin and bacterial load.

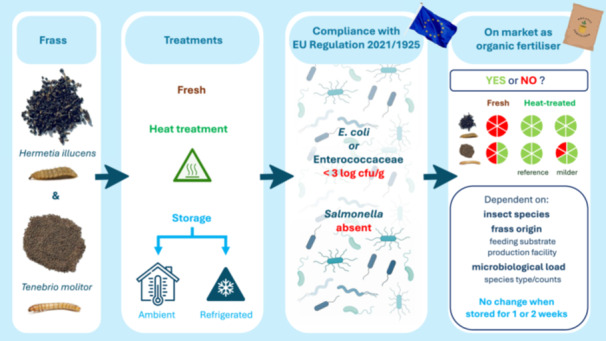

## 1 Introduction

Driven by the rising demand for safe, nutritious, and sustainably produced feed and food, insect farming has become a growing industry. European production, which mainly focuses on rearing insects for the animal feed market (aquafeed, pig, poultry, and pet feed), was estimated at 11,000 tonnes in 2023 and is predicted to increase 10–60 times by 2030. Black soldier fly larvae (BSFL) (*Hermetia illucens* L.), yellow mealworms (YM) (*Tenebrio molitor* L.), and house crickets (*Acheta domesticus* L.) are the most reared species (IPIFF 2023). To achieve the estimated growth, the rearing process, in which insects convert low‐value organic substrates into high‐value end products, will require optimization in terms of resource efficiency, product valorization, and environmental impact reduction.

EU Regulation (EC) No. 1069/2009 considers reared insects as farmed animals, which implies they can only be grown on substrates from plant origin (EC 2009). Of the 57 million tonnes of food waste generated in the EU in 2020, 30% was suited for insect bioconversion into protein, but partly due to legal restrictions, only 3% was used (Lalander and Vinnerås 2022). Broadening the range of approved feed substrates, including former foodstuffs containing meat and fish, will be required to expand insect production (IPIFF 2020). This will decrease the amount of currently unexploited biomass whilst boosting the volume of generated insect rearing products and by‐products. To reduce costs and enhance the sustainability and circularity of insect farming, these by‐products must be valorized. Lipids, dead insects, shed exoskeletons, and chitin may be used as biofuel, in cosmetics, animal/human medicine, and agriculture (Chavez and Uchanski 2021).

Frass, the major rearing by‐product is a mix of feeding substrate, excrements, and parts of farmed insects, dead eggs, and less than 3 weight % of dead insects. Depending on the nature of the substrate, between 200 and 600 kg of wet frass is generated from 1000 kg of this starting material (Lopes et al. 2022). As frass is rich in nutrients and microorganisms, studies suggest it may have potential as soil amendment (improve physical properties and microbiological composition), plant bio‐fertilizer (direct via nutrient availability or indirect through soil improvement) and biostimulant (presence or stimulation of plant growth‐promoting rhizobacteria, improvement of plant defence) (Barragán‐Fonseca et al. 2022). On the other hand, high numbers of Enterobacteriaceae (4.0–9.5 log colony forming units [cfu]/g), lactic acid bacteria (5.0–9.2 log cfu/g), and bacterial endospores (4.0–7.6 log cfu/g) have been reported in frass (Gold et al. 2020; Osimani et al. 2018, 2019, 2021; Wynants, Frooninckx, Crauwels, et al. 2019). Several species of these families are potential human pathogens such as the enterobacteria *Salmonella* and *Escherichia coli*, lactic acid bacteria from the genus Enterococcaceae, and the spore‐forming bacteria *Bacillus cereus* and *Clostridium perfringens*. More specifically, *E. coli* O157:H7 and *S. enterica* have been linked to disease outbreaks after consumption of fresh vegetables. Pathogen transfer may occur through the application of contaminated irrigation water, manure, or compost. Both pathogens have very low infectious doses (10–100 cells) and can persist for extended periods in manure, compost, and in manure/compost‐amended soils (Fatica and Schneider 2011; Franz and Van Bruggen 2008). They even survive in the rhizosphere of vegetable plants, on plant leaves and may invade plant tissues (Ongeng et al. 2015). Their potential presence in (frass) fertilizers poses health risks, not only to farmers during application in plant cultivation but also to consumers.

To mitigate these risks, Regulation (EU) 2021/1925 sets standards for production and placing on the market of insect frass as an organic fertilizer and/or soil improver, aligned with those applicable to processed animal manure. The requirements include that frass should be subjected to a heat treatment process of at least 70°C for at least 60 min and to a reduction in spore‐forming bacteria, where they are identified as a relevant hazard. Representative samples taken during or immediately after processing must comply with the microbiological criteria shown in Table 2 (EC 2021). After application of this reference treatment on BSFL frass, Van Looveren et al. (2022) observed that both the number of Enterobacteriaceae (7 log cfu/g) and vegetative *Clostridium perfringens* (inoculated at 5 log cfu/g) were reduced to a value below the detection limit of 1.0 log cfu/g, while *Salmonella* spp. (inoculated at 5 log cfu/g) were no longer detected in 25 g of frass. They concluded that processed BSFL frass may be marketed as organic fertilizer and/or soil improver. It remains, however, necessary to investigate compliance with the EU criteria stipulated in Table 2, for both BSFL and YM frass, not only immediately after the reference heat treatment but also after subsequent storage. While the thermal treatment process minimizes the risks of pathogen transfer when frass is used in agriculture, it may at the same time compromise its fertilizer potential due to alterations in nutrient and biostimulant profiles and loss of plant health‐promoting bacteria. It will therefore be essential to develop treatment procedures that mitigate health risks but at the same time preserve the chemical and beneficial microbiological properties of the frass (Lopes et al. 2022). Promising in this perspective is the observed minimal reduction of total viable counts by Van Looveren et al. (2022) after reference heat treatment of BSFL frass. The effect of milder treatments on the microbial load of BSFL and YM frass remains to be investigated. Assessment of a specific microbiological risk profile for insect frass, which may differ from other types of organic fertilizers, may be appropriate and may even open perspectives on the use of untreated frass in agriculture, thereby further improving the insect sectors sustainability. Considering the desired diversification of authorized insect feeding substrates, microbiological risk assessment of frass will become increasingly important. Compared to using plant‐based wastes as substrates, using food and household wastes for insect rearing may increase the microbial load of the residual frass, as well as the health risks on its application in agriculture.

The aim of this study was (1) to examine the impact of the legally imposed heat treatment at 70°C for 60 min (reference treatment) and to evaluate the effect of alternative milder treatments (lower temperatures and/or shorter times) on the microbiological quality and safety of frass from BSFL and YM rearing, with focus on Enterobacteriaceae count reduction. Next (2) to investigate the efficacy of both the reference and the most effective alternative treatment using *Salmonella* spp.‐inoculated frass. Finally (3) to assess the effect of storage, both refrigerated (4°C) and at ambient temperature (20°C–25°C) on the microbiological quality/safety of fresh and heat‐treated frass, with focus on pathogens (*E. coli*, Enterococcaceae, and *Salmonella*) mentioned in EU Regulation 2021/1925.

## 2 Materials and Methods

**Figure 1:**
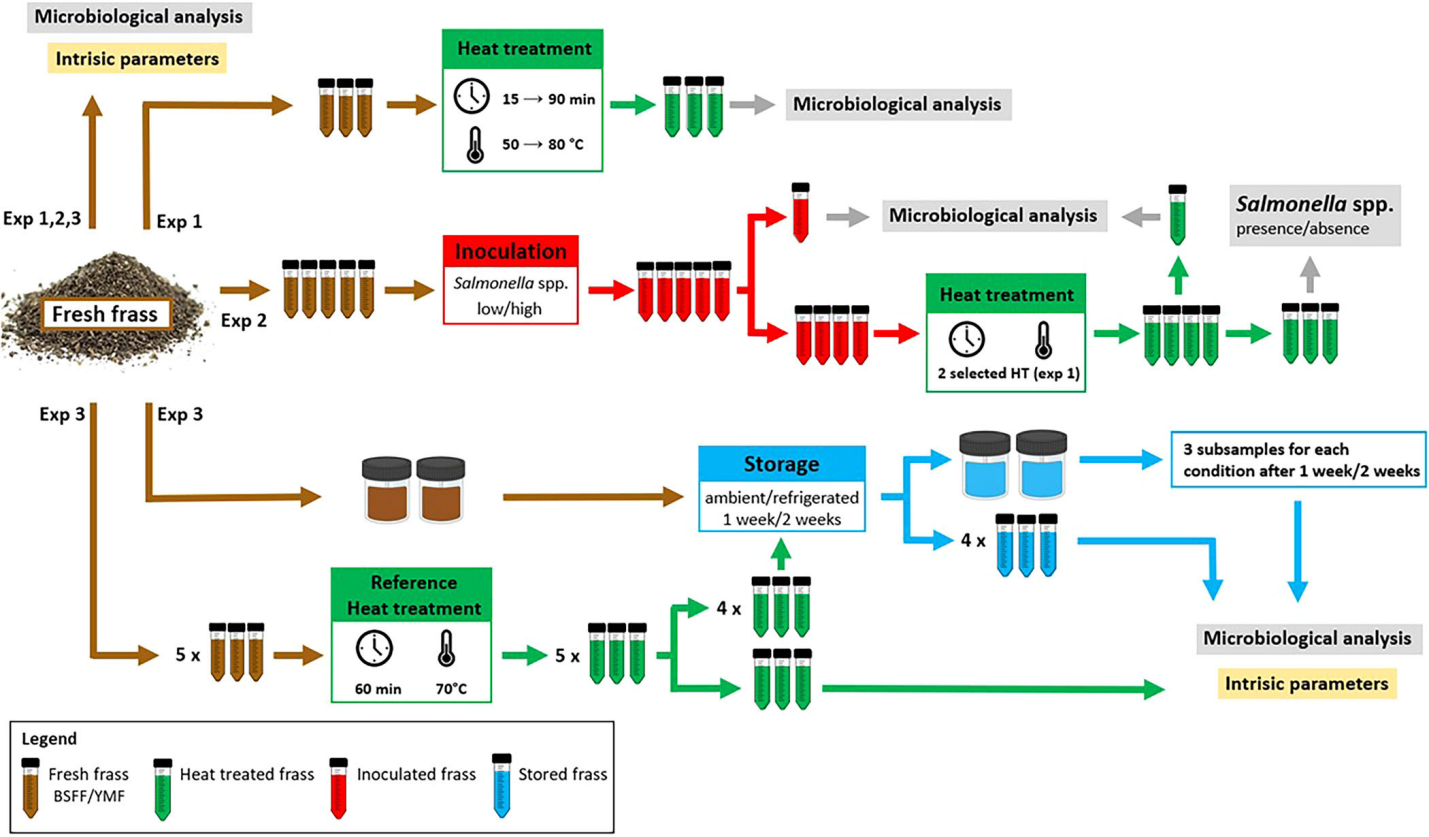
Schematic representation of experiments (Exp) 1, 2, and 3 for frass samples (BSFL or YM); heat treatment (green), *Salmonella* spp. inoculation (red), storage (blue), and performed analyses (yellow and gray).

Three experiments were conducted to assess the microbiological quality and safety of BSFL and YM frass. In the first experiment, fresh frass was processed at different temperature/time combinations. In the second experiment, *Salmonella* spp.‐inoculated frass was subjected to both the reference and milder heat treatments. In the last experiment, fresh and heat‐treated frass was stored, refrigerated, and at ambient temperature, for 1 and 2 weeks. A schematic representation of experiments 1, 2, and 3 and associated analyses is presented in Figure 1.

### 2.1 Frass Sources

The frass used in the first experiment of this study originated from pilot scale rearing of YMs (*Tenebrio molitor*, code TM) and BSFL (*Hermetia illucens*, code HI). Samples HI1 and TM1 were delivered by the Centre of Expertise Sustainable Biomass and Chemistry (Thomas More University of Applied Sciences, Geel, Belgium), samples HI2 and TM2 by the Insect Research Centre of Inagro (Rumbeke‐Beitem, Belgium). The frass used in the *Salmonella* spp. inoculation experiment (HI3‐4 and TM3‐4) originated from four different industrial scale insect rearing facilities. The storage experiment was conducted on frass from pilot scale rearing by the Centre of Expertise Sustainable Biomass and Chemistry (HI5‐6 and TM5‐6). Table 1 provides an overview of conducted experiments and corresponding samples used. Frass was collected immediately after harvesting the larvae. Without any pretreatment, it was shipped to the laboratory and stored at 4°C. Detailed information on the nature and composition of the feeding substrate was not provided, but none of the substrates contained animal by‐products.

**Table 1:** Frass samples used in this study: Sample codes, insect species, origin, rearing scale, and corresponding experiments.

Sample code	Insect species	Origin	Rearing scale	Experiment number and description	
HI1	Black soldier fly	Thomas More	Pilot		**Fresh frass**
HI2	Black soldier fly	Inagro	Pilot		Heat treatment test
TM1	Yellow mealworm	Thomas More	Pilot	1	Temperatures (50°C–80°C)
TM2	Yellow mealworm	Inagro	Pilot		Durations (15–90 min)
HI3	Black soldier fly	Rearer 1	Industrial		**Fresh and** ***Salmonella*** **spp.‐inoculated frass**
HI4	Black soldier fly	Rearer 2	Industrial		Heat treatment validation
TM3	Yellow mealworm	Rearer 3	Industrial	2	Reference treatment (70°C–60 min)
TM4	Yellow mealworm	Rearer 4	Industrial		Selected alternative treatments
HI5	Black soldier fly	Thomas More	Pilot		**Fresh and reference heat‐treated frass**
HI6	Black soldier fly	Thomas More	Pilot		Storage 1 and 2 weeks
TM5	Yellow mealworm	Thomas More	Pilot	3	Refrigerated (4°C)
TM6	Yellow mealworm	Thomas More	Pilot		Ambient temperature (20°C–25°C)

### 2.2 Heat Treatment of Fresh Frass Using Multiple Temperature/Time Combinations

The effect of heat treatment on frass from BSFL and YM rearing was examined by submitting four frass samples (HI1‐2 and TM1‐2) to a series of heat treatments using different temperature/time combinations following the protocol described by Van Looveren et al. (2022). Heat treatments ranged in temperature from 50°C to 80°C and in time from 15 to 90 min. A total of 20 tests were conducted, each consisting of one heat treatment (at a specific time/temperature combination) of three 15 g subsamples of one frass sample. For each test, four Sarstedt conical tubes (50 ml) were filled with 15 g frass, capped, and submerged in a temperature‐monitored hot water bath (WNB14, Memmert, Schwabach, Germany). One tube was used for temperature monitoring using a datalogger (Escort Junior High Temperature Logger HJ‐FP‐V‐16‐CI, Escort Messtechnik AG, Aesch, Switzerland). When the treatment temperature was reached at the central point of this tube, the samples were kept in the water bath during the desired treatment time. After heat treatment, the samples were cooled in an ice bath for 30 min. Intrinsic parameters (see Section 2.7) were determined in triplicate for fresh frass samples. The efficacy of the heat treatment was assessed by comparison of microbiological parameters (Total Viable Count [TVC] and Enterobacteriaceae count, see Section 2.6), determined in triplicate, before and after frass treatment. TVC was used to assess the overall microbial load in frass samples, while Enterobacteriaceae served as indicator organisms to evaluate the effectiveness of heat treatment in reducing *Salmonella*, which belongs to this bacterial family. To align Enterobacteriaceae count with the legal criterion for *Salmonella* (absence in 25 g), heat treatment was considered effective if Enterobacteriaceae reduction below the detection limit of 1.0 log cfu/g was achieved.

### 2.3 Heat Treatment of *Salmonella* spp.‐Inoculated Frass

To evaluate the specific *Salmonella* spp. count‐reducing potential of heat treatment, in a second experiment, four frass samples (HI3‐4 and TM3‐4) were each inoculated with *Salmonella* spp. at two levels (see Section 2.5) and submitted to two treatments, the reference treatment (70°C–60 min) and a milder alternative treatment, selected based on results of the first experiment (see Section 2.2). Intrinsic parameters, Enterobacteriaceae count, and presence/absence of *Salmonella* spp. in 25 g were first determined in triplicate for the untreated, uninoculated frass. Next, for each frass sample (HI3‐4 and TM3‐4), each heat treatment and each inoculation level (low and high), five subsamples of *Salmonella* spp.‐inoculated frass were prepared (see Section 2.5). One subsample was used to determine Enterobacteriaceae and *Salmonella* spp. counts in the untreated inoculated frass (inoculation check). Four subsamples were submitted to heat treatment as described in Section 2.2. One was then used to determine Enterobacteriaceae count and three to assess the presence/absence of *Salmonella* spp. after heat treatment.

### 2.4 Refrigerated and Ambient Temperature Storage of Fresh and Reference Heat‐Treated Frass

The third experiment was conducted to evaluate the effect of storage, both refrigerated (4°C) and at ambient temperature (20°C–25°C), on the intrinsic and microbiological properties of fresh and reference heat‐treated frass, with focus on the pathogens mentioned in EU legislation (Table 2). Intrinsic parameters and microbial counts (TVC, Enterobacteriaceae, aerobic endospores, *E. coli*, Enterococcaceae, and *Salmonella* spp., see Section 2.6) were determined in triplicate for four frass samples (HI5‐6 and TM5‐6). All samples were first analyzed fresh. Next, two subsamples of each fresh frass sample were placed in closed containers, one was stored under environmental conditions in the lab, and the other was cooled (refrigerator temp. set at 4°C). Storage temperature was measured at regular intervals using a temperature logger (Escort iLog EI‐IN‐D‐32‐L, Escort Messtechnik AG, Aesch, Switzerland). After one and two weeks of storage, three subsamples from each container were analyzed for intrinsic parameters and microbial counts. Finally, for each fresh frass sample, 15 subsamples were submitted to heat treatment (70°C–60 min) conducted according to the protocol described in Section 2.2. Of these subsamples, three were analyzed immediately after treatment, the rest were stored, six at ambient temperature and six refrigerated. Three samples of both storage conditions were analyzed after 1 week and the remaining three after 2 weeks. A schematic representation of Experiments 1, 2, and 3 and associated analyses for frass samples is presented in Figure 1. The specific frass samples used in each one of the experiments are indicated in Table 1.

### 2.5 *Salmonella* spp. Cultivation and Frass Inoculation

For the inoculation of frass samples with *Salmonella* spp., a fresh inoculation suspension (IS) was prepared daily, starting from two *Salmonella* cultures, *Salmonella enterica* subsp. *enterica* serovar Infantis (LMG 18746) and *Salmonella enterica* subsp. *enteric*a serovar Typhimurium (LMG 18732), both obtained from the Belgian Coordinated Collection of Microorganisms (BCCM, Ghent, Belgium). To avoid problems with overgrowth of nonspecific background bacteria in *Salmonella* detection, kanamycin‐resistant *Salmonella* strains were generated from these original strains as described by De Smet et al. (2021). The kanamycin‐resistant species were both cultivated overnight at 37°C in lysogeny broth (10 g/L tryptone, 5 g/L yeast extract, 10 g/L NaCl, VWR, Leuven, Belgium), supplemented with 50 µg/mL kanamycin (Thermo Fisher Scientific, Merelbeke, Belgium). The strain cultures were centrifuged at 3400*g* for 10 min and after discarding the supernatant, the pellet was diluted with peptone physiological salt solution (PPS, 0.85% NaCl, 0.1% peptone, Biokar Diagnostics, Beauvais, France) to a density of 6 McFarland units (DEN‐1 McFarland 166 Densitometer, Grant instruments, Cambridge, UK). A 1/1 mixture of both inoculum dilutions was used as IS. The *Salmonella* spp. concentrations of two separate inoculation solutions, determined in a preliminary test, were approximately 8 and 5 log cfu/mL.

Although EU regulation states that the presence/absence of *Salmonella* should be assessed for 25 g subsamples, for practical purposes, the heating experiments on inoculated frass were performed on 10 g subsamples. To avoid homogeneity problems in the inoculated frass, each Sarstedt 50 mL conical tube containing 10 g fresh frass was inoculated individually. To minimally influence the moisture content and water activity (a_w_) of the frass samples, a maximum of 800 µL IS solution was used to inoculate 10 g of frass, resulting in a maximum *Salmonella* spp. concentration of 7 log cfu/g, referred to as inoculation level high. For the low‐level inoculation, 80 µL IS was added to 10 g frass, resulting in a minimum concentration of 4 log cfu/g, referred to as inoculation level low. The *Salmonella* spp. count of each freshly prepared IS was determined on RAPID'*Salmonella* agar (Bio‐Rad Laboratories, Temse, Belgium; certified NP validation according to ISO 16140, validated by AOAC International) after 48 h incubation at 37°C. One inoculated sample was used in total for Enterobacteriaceae and *Salmonella* spp. enumeration. Four inoculated samples were heat‐treated and used in total to determine Enterobacteriaceae counts (1 tube) and presence/absence of *Salmonella* spp. (3 tubes).

### 2.6 Microbiological Analysis

Microbial counts (except for *Salmonella* spp.) were determined in triplicate on frass samples according to the ISO standards assembled by Dijk et al. (2015). Five grams of frass was used to prepare a primary dilution (1/10) in sterile PPS in a Stomacher bag. After homogenization for 60 s in a Bagmixer (Interscience, SaintNom, France), a tenfold dilution series was made in PPS and 1 mL of each dilution was plated in twofold on different media using the pour‐plate technique. TVC was determined on Plate Count Agar (PCA, Biokar Diagnostics) after incubation at 30°C for 72 h. Enterobacteriaceae count was assessed on Violet Red Bile Glucose agar (VRBG, Biokar Diagnostics) after incubation at 37°C for 24 h. Enterococcaceae count, more specific Enterococci count, was determined on Kanamycin esculin Azide Agar (KAA, Millipore, Darmstadt, Germany) after 48 h at 37°C, and *E. coli* count on Tryptone bile‐x Glucuronide Agar (TBX, VWR, Leuven, Belgium) incubated at 44°C for 24 h. For count of aerobic endospores, the primary dilution was submitted to a heat shock (80°C for 10 min) followed by pour‐plating a tenfold dilution series on PCA and incubation at 37°C for 24 h. *Salmonella* spp. count was determined by spread‐plating 100 µL of a dilution series, prepared from 5 g frass as described above, in twofold on RAPID'*Salmonella* agar (Bio‐Rad Laboratories) and incubation at 37°C for 24 h. For the enumeration of *Salmonella* spp. in the inoculated frass samples, the agar was supplemented with kanamycin (50 µ/mL agar). Presence/absence of *Salmonella* was determined following the RAPID'*Salmonella* short protocol (Bio‐Rad Laboratories). For fresh frass, the normal protocol (25 g sample intake) was used. For inoculated frass after heat treatment, the protocol was adapted to 10 g sample intake. A selective RAPID'*Salmonella* capsule (Bio‐Rad Laboratories) was added to 250 mL buffered peptone water (Bio‐Rad Laboratories). This solution was used to prepare a 1/10 sample dilution, which was incubated for selective enrichment at 42.5°C for 16 to 22 h. Next, 10 µL of the enriched dilution was spread‐plated in twofold on RAPID'*Salmonella* agar supplemented with 50 µg/mL kanamycin and incubated at 37°C for 48 h. To verify the RAPID'*Salmonella* short protocol adapted to smaller sample intake, 10 g YM frass was inoculated (5 log cfu/g) and analyzed for the presence/absence of *Salmonella* spp. The test was performed in triplicate, and *Salmonella* spp. were detected in all replicates, confirming the validity of the adapted test protocol.

### 2.7 Determination of Intrinsic Properties (Moisture Content, Water Activity, and pH)

Frass moisture content (%) was calculated from the weight difference of a 5 g subsample before and after overnight oven‐drying (Memmert, Schwabach, Germany) at 105°C. Determination of water activity was performed using a LabMaster a_w_ meter (Novasina, Lachen, Switzerland) until measured a_w_ and temperature (25°C) were stable for at least 5 min. pH was measured at ambient temperature using a digital pH meter (pH 1100 H set, VWR, Germany) after addition of 8.5 mL demineralized water to 5 g frass, following the method described by Van Looveren et al. (2022). Intrinsic parameters were determined in triplicate.

### 2.8 Microbiological Safety Criteria

Regulation (EU) 2021/1925 standards for production and placing in the market of insect frass as an organic fertilizer and/or soil improver are shown in Table 2.

**Table 2:** Microbiological standards applying to the placing on the EU market of insect frass as fertilizer and soil improver (Commission Regulation [EU] 2021/1925).

Sampling	Micro‐organism	Sample number	Criteria		
			c	m	M
During or immediately after processing	*Escherichia coli*	5	5	0	1000
	**OR**				
	Enterococcaceae	5	5	0	1000
During or on withdrawal from storage	*Salmonella*	5	0	0	0
			Absence in 25 g		

*Note:* c = number of samples for which the bacterial count in cfu/g may be between m and M.

m = threshold value (lower limit) for the number of bacteria.

M = maximum value (upper limit) for the number of bacteria, if the bacteria number for one sample exceeds M, the batch is considered insufficiently processed, 1000 cfu/g = 3 log cfu/g.

### 2.9 Statistical Analysis

All statistical analyses were performed using JMP Pro 16.0.0 software package from SAS, considering a significance level of 0.05. One‐way analysis of variance (ANOVA), followed by a Tukey HSD post‐hoc test, was used to compare intrinsic parameters as well as microbial plate counts before and after heat treatment. For microbial counts below the detection limit, the respective detection limit itself (1 or 2 log cfu/g) was used.

## 3 Results and Discussion

### 3.1 Intrinsic Parameters of Fresh Frass

Results for moisture content, water activity, and pH for the fresh frass samples are presented in Table 3. The average moisture content for BSFL frass (37.7 ± 6.5%; range 29%–45%) was significantly higher compared to the average for YM frass (13.7 ± 2.4%; range 11%–17%) (*p* < 0.0001). The moisture content results are in accordance with previously reported values. Wynants, Frooninckx, Crauwels, et al. (2019) measured values between 23% and 74% for the residue of BSFL reared at different facilities on vegetable waste streams. A value of 36% moisture was found by Van Looveren et al. (2021) for frass from BSFL raised on a similar substrate. In a study on YM frass from four different insect rearers, moisture contents between 10% and 19% were reported (OVAM 2022).

**Table 3:** Moisture content (%), water activity (a_w_), and pH for fresh black soldier fly larvae frass (HI) and yellow mealworm frass (TM). Results represent the mean and standard deviation of three replicates.

	Moisture content (%)	Water activity (−)	pH (−)
HI1	29.4 ± 2.1^d^	0.92 ± 0.00^d^	n.d.
HI2	41.8 ± 0.8^b^	0.96 ± 0.00^b^	n.d.
HI3	44.7 ± 0.3^a^	0.97 ± 0.00^a^	n.d.
HI4	44.3 ± 0.3^a,b^	0.96 ± 0.00^b^	n.d.
HI5	35.3 ± 0.0^c^	0.94 ± 0.00^c^	7.8 ± 0.0^a^
HI6	30.7 ± 0.0^d^	0.94 ± 0.00^c^	7.6 ± 0.0^b^
TM1	11.4 ± 0.1^c,d^	0.60 ± 0.01^e^	n.d.
TM2	10.7 ± 0.4^d^	0.61 ± 0.00^e^	n.d.
TM3	14.0 ± 0.1^b^	0.68 ± 0.00^c^	n.d.
TM4	16.7 ± 0.9^a^	0.77 ± 0.01^b^	n.d.
TM5	12.6 ± 0.1^c^	0.64 ± 0.00 ^d^	6.1 ± 0.0^a^
TM6	16.3 ± 0.2^a^	0.80 ± 0.01^a^	6.2 ± 0.0^b^

*Note:* a–f: Means of samples for either HI or TM with the same letter in superscript within a column do not differ significantly (*p* ≥ 0.05).

Abbreviation: n.d., not determined.

The average water activity measured for BSFL frass was 0.94 ± 0.02, while the average YM frass value of 0.67 ± 0.08 was lower. Contrary to the moisture content range, BSFL frass showed a limited a_w_ range from 0.92 to 0.97, while the range for YM frass, a_w_ 0.60–0.80, was broader. The measured water activity for BSFL frass is in accordance with previously reported values. Wynants, Frooninckx, Crauwels, et al. (2019) measured a_w_ between 0.83 and 0.98 for BSFL reared on organic waste streams, and a value of 0.97 was reported by Van Looveren et al. (2021, 2022). The lower a_w_ value found for YM frass is in accordance with the value of 0.57 reported by Cesaro et al. (2022). While moisture content indicates the total amount of water present in a product, the a_w_ value reflects the moisture available for microbial growth. In a low a_w_ environment, microorganisms become dormant, and generally at a_w_ below 0.60, no microbial proliferation can occur. For *E. coli*, an a_w_ below 0.96 is considered to inhibit growth, minimal a_w_ values of 0.93 and 0.96 are reported for *Salmonella* growth (Preetha and Narayanan 2020; Rolfe and Daryaei 2020), and an a_w_ below 0.92 prevents Enterococcaceae proliferation (Balamurugan et al. 2014). All water activity values for frass samples analyzed in this study are above the general microbial growth limit (a_w_ < 0.6) even though only slightly for YM frass. Growth of most microorganisms will therefore be restricted or inhibited in the YM frass, while in BSFL frass, proliferation may be possible.

Average pH values for fresh frass were 7.8 ± 0.0 and 7.6 ± 0.0 for HI5 and HI6, and significantly lower values of 6.1 ± 0.0 and 6.2 ± 0.0 were found for TM5 and TM6 (*p* < 0.0001). Van Looveren et al. (2022) reported pH 7.2 for frass from BSFL raised on chicken feed, Setti et al. (2019) found a higher pH value of 8.8 when a mix of wheat bran, alfalfa, and corn meal was used as substrate and Song et al. (2021), who used a mix of okra and wheat, reported pH 7.8 for the resulting frass. Lower pH values between 5.6 and 7.4 were reported by Gold et al. (2020) and Kawasaki et al. (2020) for frass from BSFL raised on canteen/household waste. Peng et al. (2020) reported a pH value of 7.5 for frass from YM raised on wheat bran, a lower average pH of 6.3 was reported for four YM frass samples from different rearers (OVAM 2022). The wide range of frass pH values observed in these studies suggests variability depending on both the substrate and the insect species for this intrinsic factor. While microbial growth is optimal at pH 7, growth is possible in a pH range between 5 and 9 but limited below pH 6 (Balamurugan et al. 2014). Some species of *E. coli* and *Salmonella*, however, tolerate a pH as low as 4.4 and 4.2, respectively (Morasi et al. 2022). The near‐neutral pH of the fresh BSFL frass (HI 5‐6) will allow growth of most microbes, while the slightly more acidic nature of the YM frass (TM 5‐6) may restrict proliferation of certain microorganisms.

### 3.2 Microbiological Quality/Safety of Frass Before and After Heat Treatment Using Multiple Temperature/Time Combinations

**Figure 2:**
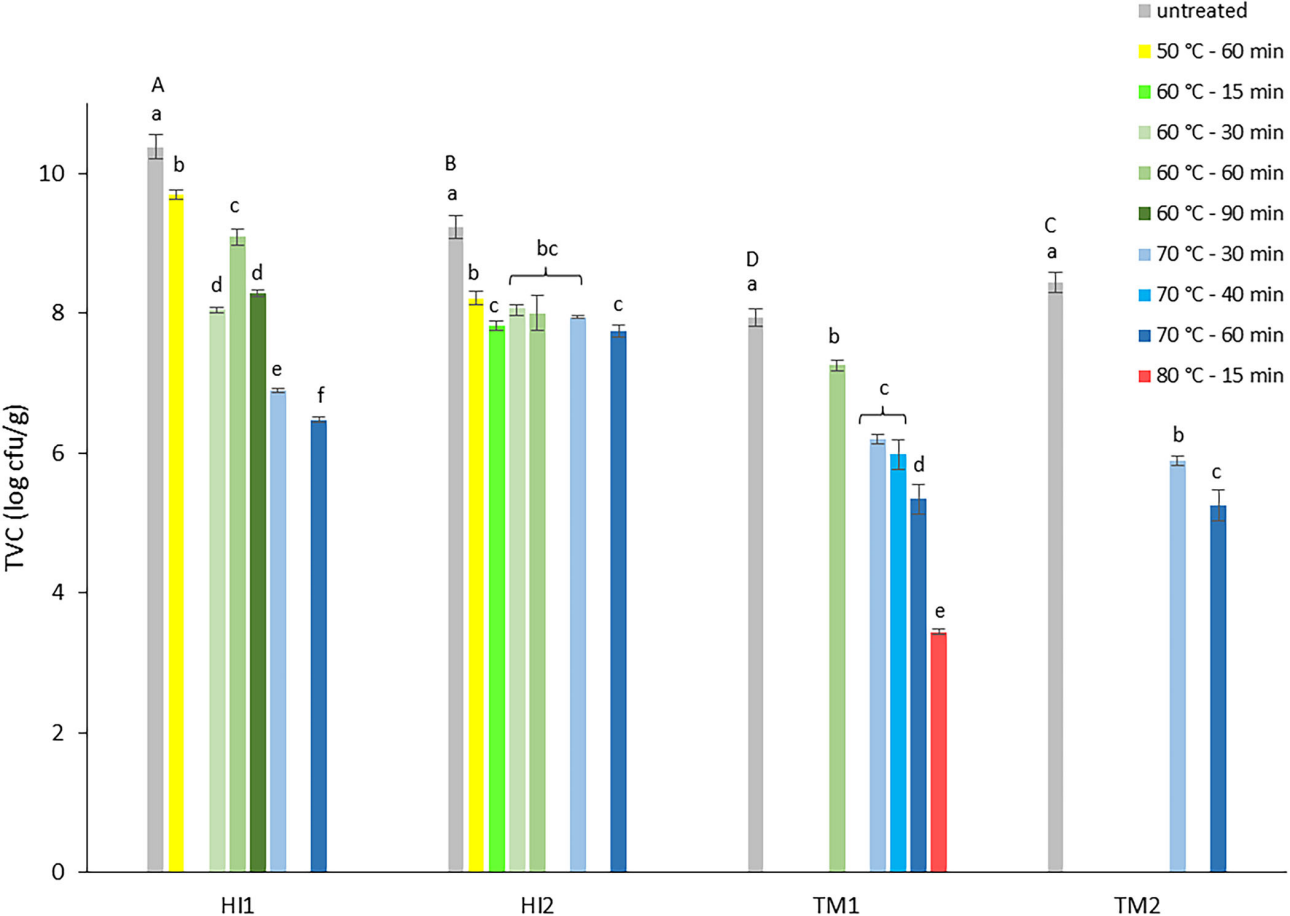
Results for total viable count (log cfu/g) for black soldier fly larvae frass (HI1‐2) and yellow mealworm frass (TM1‐2) before (untreated samples, gray) and after heat treatment at different temperature/time combinations, sorted from lowest (50°C, yellow) to highest (80°C, red) applied temperature; reference treatment (70°C–60 min), dark blue. No bar indicates time/temperature combination not applied to a specific frass sample. Data are the mean value and standard deviation of three replicates. a–f: means of samples with the same letter in superscript per frass sample do not differ significantly (*p* ≥ 0.05). A–D: Means of untreated frass samples with the same letter in superscript do not differ significantly (*p* ≥ 0.05).

**Figure 3:**
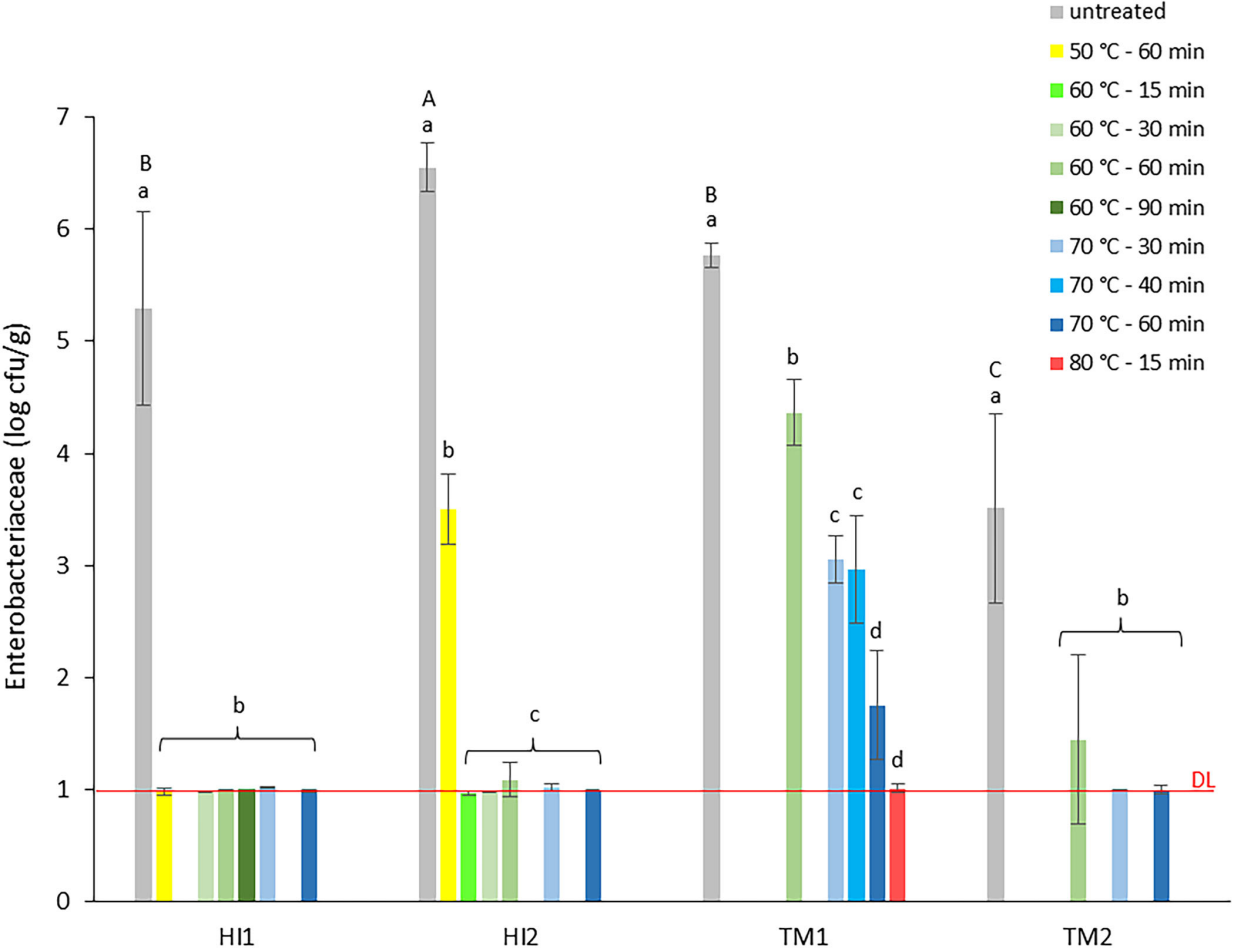
Results for Enterobacteriaceae count (log cfu/g) for black soldier fly larvae frass (HI1‐2) and yellow mealworm frass (TM1‐2) before (untreated samples, gray) and after heat treatment at different temperature/time combinations, sorted from lowest (50°C, yellow) to highest (80°C, red) applied temperature; reference treatment (70°C–60 min), dark blue. No bar indicates a time/temperature combination not applied to a specific frass sample. Data are the mean value and standard deviation of three replicates. DL: detection limit, 1 log cfu/g. a–d: means of samples with the same letter in superscript per frass sample do not differ significantly (*p* ≥ 0.05). A–C: means of untreated frass samples with the same letter in superscript do not differ significantly (*p* ≥ 0.05).

The first experiment examined the impact of heat treatment on BSFL frass (HI1‐2) and YM frass (TM1‐2), with a focus on TVC and Enterobacteriaceae count reduction. Counts before and after heat treatments are presented in Figure 2 (TVC) and Figure 3 (Enterobacteriaceae).

The microbial load of the fresh frass samples was high, with TVC values of 10.4 ± 0.2 and 9.2 ± 0.2 log cfu/g for BSFL frass samples HI1 and HI2, and lower values of 7.9 ± 0.1 and 8.4 ± 0.1 log cfu/g for YM frass samples TM1 and TM2. Enterobacteriaceae count for fresh HI1 was 5.3 ± 0.9 log cfu/g, significantly lower than the HI2 value of 6.5 ± 0.2 log cfu/g (*p* = 0.0146). YM frass sample TM1 had an Enterobacteriaceae count of 5.8 ± 0.1 log cfu/g, comparable to those of HI frass, while for TM2 a significantly lower count of 3.5 ± 0.8 log cfu/g was found (*p* < 0.001). The measured high TVCs and Enterobacteriaceae counts in fresh BSFL frass are in accordance with TVC values between 8.5 and 10.2 log cfu/g reported by Wynants, Frooninckx, Crauwels, et al. (2019) and 9.2 log cfu/g by Van Looveren et al. (2022) for larvae raised on similar substrates. Their reported Enterobacteriaceae counts were between 5.7 and 9.5 log cfu/g and 7.1 log cfu/g, respectively. Data from a study on seven BSFL frass samples from six insect rearers showed Enterobacteriaceae counts between 4.5 and 7.1 log cfu/g (OVAM 2022). In the same study, values between 4.7 and 6.5 log cfu/g were found for eight YM frass samples, while Osimani et al. 2018 reported slightly higher values of 6.8 and 7.0 log cfu/g. Enterobacteriaceae count in TM1 was comparable to the reported values, while for TM2, the count was lower.

The TVC reflects the general microbial load. A value of 6–8 log cfu/g is considered normal for fresh raw vegetables and this number will rapidly increase upon spoilage and decay (Health Protection Agency 2009). Since vegetable waste streams are used as substrate in insect larvae rearing, high TVC counts in the residual frass were expected. Enterobacteriaceae are abundantly found in water, soil, and on vegetation. Burns et al. (2015) reported Enterobacteriaceae counts above 4 log cfu/g in raw materials for pig feed production (cereals, vegetable proteins, and by‐products of oil extraction). Enterobacteriaceae belong to the insect gut microbiota and will be transferred to the feeding substrate via excrements during the rearing process (De Smet et al. 2018). The Enterobacteriaceae family consists of facultatively anaerobic gram‐negative bacteria and includes harmless species as well as potential pathogens such as *Klebsiella*, *Enterobacter*, *Citrobacter*, *Salmonella,* and *Shigella* species, as well as *E. coli* (Baylis et al. 2011). The relatively high number of Enterobacteriaceae found in BSFL and YM frass might predict high contamination with potential pathogens.

Applied heat treatments reduced TVC in both frass types. Reduction varied between 0.6 and 3.9 log units. The lowest residual count was 5.0 log cfu/g. Reduction increased with higher temperature/longer time of the applied treatment procedure. Limited influence on TVC was also observed by Van Looveren et al. (2022) in their study on the impact of heat treatment (70°C–60 min) on BSFL frass. They reported an average 0.8 log unit reduction for TVC, which is less than the average reductions of 3.9 and 1.5 log units found for HI1 and HI2, respectively, for the same heat treatment in experiment 1. Praeg and Klammsteiner (2024) also observed low reduction of the TVC count in both BSFL (9.1 log cfu/g, 0.5 log unit reduction) and YM frass (5.9 log cfu/g, 0.2 log unit reduction) after 1 h oven drying at 70°C. Comparison data were not found for treatment at other time/temperature combinations. The high residual TVC does not inherently imply a safety risk when using insect frass as organic fertilizer, but suggests that, when present in frass, potential plant growth‐promoting microorganisms may survive heat treatment. Even though Praeg and Klammsteiner (2024) detected no microbial activity in fresh and heat‐treated YM frass, they did observe a significant increase in microbial abundance and activity in frass‐amended soil (12% addition). The soil microbiome boost was believed to result from a combination of nutrient input and revival of bacteria in the YM frass, even after heat treatment, by increased water availability in the soil environment.

As shown in Figure 3, all applied heat treatments significantly reduced Enterobacteriaceae counts in BSFL and YM frass. Heat treatment at 70°C as well as at 60°C, for all tested durations (90, 60, 30, and 15 min), resulted in Enterobacteriaceae counts for BSFL frass < 1.0 log cfu/g. Heat treatment at a lower temperature of 50°C was insufficient for HI2 since Enterobacteriaceae counts were only reduced to 3.5 log cfu/g. Reduction of the initial Enterobacteriaceae count of 7.1 log cfu/g in BSFL frass to a value below the detection limit, after heat treatment at 70°C for 1 h was also observed by Van Looveren et al. (2022). For YM frass TM1 the initial Enterobacteriaceae count of 5.8 log cfu/g was reduced to 1.8 log cfu/g after reference heat treatment, but for reduction below detection limit a minimum temperature of 80°C was required. For TM2, the initial Enterobacteriaceae count of 3.5 log cfu/g was about 2 log units lower compared to TM1. Treatment at 70°C for 60 as well as 30 min, reduced Enterobacteriaceae counts in TM2 below the detection limit, but at 60°C counts were only reduced to 1.4 log cfu/g.

The results indicate different responses to heat treatment, not only for frass from the rearing of different insect species (BSFL or YMs), but also for frass from the same species originating from different rearing facilities. This may not be surprising given that (a) several studies mention the intrinsic, chemical and microbiological properties of frass to be strongly related to insect species, nature and composition of the feeding substrate and rearing process (Gärttling and Schulz 2021) and (b) the inactivation of microorganisms by physical heat treatment to be influenced by water activity, moisture content, nature and composition of the product and type of bacterial strain (Syamaladevi et al. 2016). Thermal treatment for pathogen inactivation is most studied in food, where distinction is made between high‐ and low‐moisture food (HMF and LMF) based on water activity. An a_w_ ≤ 0.85 defines LMF and is considered a factor that limits bacterial growth and increases thermal resistance (Liu et al. 2022). The studied BSFL frass can be considered to have the properties of HMF (a_w_ = 0.94) while the YM frass can classify as LMF (a_w_ = 0.60). The difference in a_w_ and % moisture may explain why Enterobacteriaceae count reduction in BSFL frass after heat treatment at a similar time–temperature is more outspoken than reduction in YM frass. However, this does not clarify the different responses of both YM frass samples, with similar moisture content (11%) and a_w_ (0.6) values. The higher Enterobacteriaceae count for the fresh TM1 (5.8 log cfu/g) compared to TM2 (3.5 log cfu/g) may justify the observed responses.

In this experiment, Enterobacteriaceae were used as indicator organisms for *Salmonella* to evaluate pathogen reduction in insect frass by heat treatment. A decrease in counts below the detection limit (1.0 log cfu/g) was set as a criterion for success. However, results obtained for Enterobacteriaceae may not be completely similar to those for *Salmonella,* as thermal inactivation differs between species. Further, the legal criterion for *Salmonella* (absence of the pathogen in a 25 g sample) is stricter, implying the necessity for complete elimination. Results of the first experiment indicated successful reduction of Enterobacteriaceae counts in BSFL frass at temperatures of at least 60°C for all tested times. Reduction in YM frass at reference conditions (70°C–60 min) appeared to be insufficient in case of high contamination levels as well as at temperatures below 70°C for both high and low contamination. To verify the observed responses by means of *Salmonella* spp.‐inoculated frass in the second experiment it was therefore decided (a) to evaluate *Salmonella* elimination in BSFL and YM frass after reference treatment (70°C–60 min), (b) evaluate *Salmonella* elimination in BSFL frass at lower temperature/shorter time (60°C–30 min), and (c) evaluate *Salmonella* elimination in YM frass at lower temperature/similar time (60°C–60 min).

### 3.3 Microbiological Quality of *Salmonella* spp.‐Inoculated Frass Before and After Selected Heat Treatments

In the second experiment, the reduction of bacterial counts after heat treatment was evaluated by means of *Salmonella* spp.‐inoculated BSFL and YM frass. Enterobacteriaceae counts for fresh as well as for untreated and heat‐treated *Salmonella* spp.‐inoculated frass are presented in Table 4. Enterobacteriaceae counts for fresh YM frass were similar, 5.6 ± 0.2 and 5.8 ± 0.1 log cfu/g for TM3 and TM4, respectively. BSFL frass HI3 had a significantly higher Enterobacteriaceae count of 7.1 ± 0.0 log cfu/g while HI4 had a significantly lower count of 4.1 ± 0.3 log cfu/g (*p* = 0.0196). Enterobacteriaceae results are in accordance with previously reported values as mentioned in Section 3.2 and with Experiment 1 results. *Salmonella* spp. inoculation of the frass increased Enterobacteriaceae counts, confirming successful inoculation.

**Table 4:** Results for Enterobacteriaceae counts (log cfu/g) for black soldier fly larvae (HI3‐4) and yellow mealworm (TM3‐4) fresh frass and for *Salmonella* spp.‐inoculated (low–high) frass, before (T0) and after selected heat treatments (T1‐3).

Treatment	*Salmonella*	Enterobacteriaceae (log cfu/g)			
	Inoculation	HI3	HI4	TM3	TM4
T0	No	7.1 ± 0.0^A^	4.1 ± 0.3^a,C^	5.6 ± 0.2^c,B^	5.8 ± 0.1^c,AB^
untreated	Low	7.1	6.7	6.8 ± 0.0^b^	6.5 ± 0.2^b^
	High	7.5	7.9	7.8 ± 0.0^a^	7.4 ± 0.3^a^
T1	Low	< 1.0	< 1.0 ± 0.0^b^	< 1.0	< 1.0 ± 0.0^e^
70°C–60 min	High	< 1.0	< 1.0 ± 0.0^b^	< 1.0	< 1.0 ± 0.0^e^
T2	Low	< 1.1	< 1.0 ± 0.0^b^	*	*
60°C–30 min	High	< 1.0	< 1.0 ± 0.0^b^	*	*
T3	Low	*	*	3.8 ± 0.2^d^	3.1 ± 0.1 ^d^
60°C–60 min	High	*	*	3.1 ± 0.2^e^	2.4 ± 0.3 ^d^

*Note:* Mean and standard deviation of 2 replicates or single result. *Heat treatment not conducted.

A–C: T0 uninoculated, means with the same letter in superscript do not differ significantly (*p* ≥ 0.05).

a–e: Means within a column, with the same letter in superscript do not differ significantly (*p* ≥ 0.05).

The reference heat treatment (T1, 70°C–60 min) as well as the milder treatment (T2, 60°C–30 min) significantly reduced Enterobacteriaceae counts for BSFL frass (HI3 and HI4) to a value below the detection limit, in accordance with experiment 1 results. While for YM frass reference heat treatment (T1) induced a significant reduction to a value below the detection limit, lowering the temperature to 60°C (T3) resulted in an average 4.0 log unit reduction, which was significant, but insufficient to achieve an Enterobacteriaceae count < 1.0 log cfu/g. During the first experiment, however, an average reduction of only 1.7 log units was observed for heat treatment under similar conditions. The higher moisture content of 15.4% and a_w_ of 0.74 for the YM frass, compared to values of 11.0% and 0.68 in the first experiment, may have contributed to a larger reduction.

The average *Salmonella* spp. count for five inoculation solutions, with measured turbidity of 6 MFU, was 7.5 ± 0.5 log cfu/mL. *Salmonella* was not detected in 25 g fresh frass samples before inoculation. An average low‐level count of 5.7 ± 0.8 log cfu/g and high‐level count of 6.6 ± 0.6 log cfu/g were measured in the *Salmonella* spp.‐inoculated, untreated frass (Table 5). *Salmonella* spp. were not detected after reference heat treatment (T1, 70°C–60 min) in inoculated samples of BSFL and YM frass. The alternative treatment at lower temperature/shorter time (T2, 60°C–30 min) also successfully eliminated the pathogen in all inoculated HI samples. For YM frass, however, the alternative treatment at lower temperature/similar time (T3, 60°C–60 min) eliminated *Salmonella* spp. in TM3 samples, but this was not the case in TM4 samples. In those samples, for the low and high inoculation level, the pathogen was detected in all three replicates.

**Table 5:** Results for the presence/absence of *Salmonella spp*. in 25 g untreated (T0), uninoculated black soldier fly larvae (HI3‐4) and yellow mealworm (TM3‐4) frass; results for *Salmonella* spp. counts (log cfu/g) in inoculated (low‐high), untreated (T0) frass; results for presence/absence of *Salmonella* spp. in 10 g inoculated frass after heat treatments (T1‐3).

Treatment	*Salmonella*	*Salmonella* spp. (log cfu/g)			
	Inoculation	HI3	HI4	TM3	TM4
T0	No	ND 25 g	ND 25 g	ND 25 g	ND 25 g
untreated	Low	4.9	4.6	6.3	6.1
	High	6.0	6.1	7.4	6.8
T1	Low	ND	ND	ND	ND
70°C–60 min	High	ND	ND	ND	ND
T2	Low	ND	ND	*	*
60°C–30 min	High	ND	ND	*	*
T3	Low	*	*	ND	D
60°C–60 min	High	*	*	ND	D

*Note:* ND 25 g: not detected in 25 g, 1 replicate; D/ND: detected/not detected in 10 g, 3 replicates.

* heat treatment not conducted.

Van Looveren et al. (2022) reported similar observations for BSFL frass. *Salmonella* spp.‐inoculated at 5.1 to 5.6 log cfu/g, were no longer detected after frass treatment at 70°C for 60 min. To the best of our knowledge, the effect of thermal treatment on pathogen reduction in YM frass has never been reported. Therefore, observations can only be compared to those reported for low‐moisture foods, as previously mentioned in Section 3.2. Thermal resistance of microorganisms during heat treatment in a (food) matrix is often quantified using the decimal reduction time (D‐value) which represents the time in minutes required to obtain 1 log reduction at a given temperature. The value is derived from the log‐linear model log(N/N_0_) = −t/D_T_, in which N_0_ and N (cfu/g) are the initial and survival pathogen count after treatment time t (min) at temperature T (°C) (Van Asselt and Zwietering 2006). Gu et al. (2022) studied thermal inactivation of *Salmonella* and *Enterococcus faecium* in inoculated cornmeal. They reported D‐values of 37.5 and 2.4 min for *Salmonella* spp. in cornmeal with a_w_ values of 0.76 and 0.75 at 16% and 28% moisture content, respectively, when a treatment temperature of 60°C was used. At 68°C the D‐values were reduced to 10.1 and 0.5 min, respectively. Villa‐Rojas et al. (2013) found D‐values of 24.0 and 7.0 min for *Salmonella* spp. in almond flour at 10% moisture and a_w_ 0.72 at the respective treatment temperatures of 62°C and 68°C. Considering 7 log cfu/g as the initial high‐level *Salmonella* spp. count for YM frass, based on these reported d‐values, a 7 log unit pathogen count reduction at 68°C would require 49 to 70 min, while at 60°C–62°C this time would amount to 168–260 min. These estimated times may explain why *Salmonella* spp. were no longer detected after reference treatment (70°C–60 min) in both TM3 and TM4 but are not consistent with observations for the alternative treatment (60°C–60 min). For both TM3 and TM4, the estimated time (more than 168 min) exceeds the effectively applied time; however, *Salmonella* spp. were not detected in TM3, heat treated for only 60 min.

YM frass samples TM3 and TM4 had significantly different a_w_ values (0.68 and 0.77) and moisture content (14% and 17%) (*p* < 0.0001). *Salmonella* spp. counts for inoculated TM3 samples (6.3 and 7.4 log cfu/g) were slightly higher compared to TM4 (6.1 and 6.8 log cfu/g). But, despite the lower pathogen load, higher a_w_ and moisture content, heat treatment at 60°C for 60 min was insufficient for complete inactivation of the pathogen in TM4. Besides moisture content and a_w_, several studies (Dhaliwal et al. 2021; Li et al. 2014; Liu et al. 2018) also mention product composition as a parameter influencing pathogen reduction by thermal treatment. Microenvironments within a multicomponent product matrix, carbohydrate, protein, and fat composition as well as physical state (fine/course material) should be taken into consideration. The above‐reported values for low‐moisture foods (respectively cornmeal, almond flour, and pet food pellets) may therefore not apply to YM frass, despite similar a_w_ and moisture content. Frass is a multicomponent product, consisting of non‐consumed feeding substrate, insect excrements, and exuviae. The substrate itself also varies in composition and structure between production batches and facilities. TM3 and TM4 were obtained from different industrial rearers, but information on the nature and composition of the feeding substrate was not provided. Both frass samples differed in coming up time at heat treatment. Starting from 23°C, TM3 reached the treatment temperature of 60°C in 16 min while TM4 required an extra 10 min, even though, based on the higher moisture content of the latter, the opposite may be expected. Interaction between all the above‐mentioned factors will have determined *Salmonella* spp. inactivation by heat treatment in both YM frass samples. This emphasizes the importance of setting‐up specific risk profiles and subsequent treatment methods for different frass types.

Observed findings for *Salmonella* spp.‐inoculated frass confirm the efficacy of the reference heat treatment in eliminating the pathogen. As Enterobacteriaceae counts are reduced below 1.0 log cfu/g, it may be assumed that (potentially pathogenic) *E. coli* will also be reduced below the legal limit of 3.0 log cfu/g, because the species belongs to the Enterobacteriaceae family and grows on the VRBG agar used. The tested samples of BSFL and YM frass will therefore, after thermal treatment at 70°C for 60 min, both pass the microbiological safety criteria imposed by Regulation (EU) 2021/1925, allowing use as fertilizer. Based on Experiment 1 results (Figure 3), similar conclusions can be drawn for BSFL frass after milder treatment, provided a minimum temperature–time regime of 60°C–15 min is applied. For YM frass however, Enterobacteriaceae and *Salmonella* spp. counts are reduced by heating the frass at 60°C for 1 h but insufficient to meet the legal criteria. Even though the *E. coli* count in fresh YM frass will most likely be lower than Enterobacteriaceae count, as it was not tested, no conclusion can be made for the species itself.

In this study, frass was inoculated with *Salmonella* spp. at contamination levels between 5.7 and 6.6 log cfu/g to simulate “worst case” highly contaminated frass. Most likely this is not representative for *Salmonella* contamination in frass from the insect rearing industry. None of the tested fresh frass samples contained the pathogen, and research papers on microbiological quality of BSFL and YM frass usually mention that the pathogen was not detected (De Smet et al. 2021; Osimani et al. 2018, 2019; Van Looveren et al. 2022; Wynants, Frooninckx, Van Miert et al. 2019). Yet, in a study by Wynants, Frooninckx, Crauwels, et al. (2019) on 20 BSFL frass samples originating from different rearing locations, the presence of *Salmonella enterica* serovar Agona was reported in one of three batches from the same provider. The pathogen source, either the feeding substrate or the rearing environment, could not be established. This reporting indicates that *Salmonella* spp. contamination of insect frass is possible. Moreover, since diversification of authorized substrates is considered a major factor for upscaling insect production, in the EU, in the future, insects may possibly be industrially reared on waste streams containing animal by‐products or even animal/human manure. As these substrates may contain a higher number of pathogens, including *Salmonella* and *E. coli*, compared to presently allowed substrates from plant origin, higher levels in the frass may be assumed, thereby enhancing the risks of pathogen spread upon application of frass as fertilizer/soil improver in plant cultivation after inadequate processing. In other regions in the world, current regulations are less strict, and insects may be reared on these substrates, so the risks are already present (Alagappan et al. 2022).

It should be mentioned that heat treatment in an industrial insect production setting will differ from the test procedure used in this study, in which frass was heated in closed tubes in a hot water bath, ensuring minimal influence on both moisture content and water activity. Hygienisation will be carried out differently, for example, in an oven‐setting or by application of hot air, during which both parameters will decrease, thereby influencing the efficacy of the heat treatment itself. Ensuring an effective 70°C treatment temperature in the complete batch for 1 h may be challenging. European legislation imposes “heat treatment for 1 h at 70°C,” without specifying how it should be conducted but sets specific microbiological criteria for the treated frass.

All findings emphasize the need for “tailor‐made” treatment for different frass samples, where, based on the initial microbial load and intrinsic properties, an optimal treatment procedure is developed, which ensures the frass microbiological safety, minimizes processing cost, and maximizes preservation of fertilizer and biostimulant potential. This treatment may be a heat treatment, but exploring alternatives such as composting, pelletizing, or pyrolysis should be encouraged, as well as investigating the application of untreated frass. Given the observed low contamination levels with actual food pathogens, the latter may be a valid option in specific cases as it ensures preservation of the frass nutrients and, when present, microorganisms with plant growth‐stimulating potential, without any additional cost for producers.

### 3.4 Intrinsic Properties and Microbiological Quality/Safety of Fresh and Heat‐Treated Frass Before and After Storage

Regulation (EU) 2021/1925 stipulates that, immediately after heat treatment (70°C–60 min) of frass, *E. coli* or Enterococcaceae counts should be below 3.0 log cfu/g and that *Salmonella* should be absent during or on withdrawal of frass from storage. The third experiment therefore evaluated the intrinsic and microbiological properties of fresh and heat‐treated frass as well as their evolution during storage, refrigerated (4.6°C ± 0.6°C) or at ambient temperature (27.4°C ± 0.8°C), for 1 and 2 weeks. Growth of microorganisms during frass storage may be influenced by the initial load (amount as well as type of organisms and their interactions), by intrinsic properties, nutrient profile, and matrix composition as well as extrinsic factors (temperature, relative humidity, storage environment) (Preetha and Narayanan 2020). The intrinsic parameters of fresh frass (Table 3) were minimally affected by either storage or heat treatment and storage. The pH change was limited to 0.6 units, a_w_ value changed by no more than 0.09, and subsequent change in moisture content ranged between −5% and +2%. Changes were small but often statistically significant and will therefore be discussed when relevant for observed changes in microbial parameters. Experiment 3 results for TVC and Enterobacteriaceae counts are presented in Table 6.

**Table 6:** Total Viable Count and Enterobacteriaceae counts for fresh (T0) and heat‐treated (T1) black soldier fly larvae frass (HI5‐6) and yellow mealworm frass (TM5‐6) before and after refrigerated (R) storage (C) and ambient temperature (AT) storage (A), for 1 and 2 weeks. Results represent the mean and standard deviation of three replicates.

		Total Viable Count (log cfu/g)			
Condition	Code	HI5	HI6	TM5	TM6
Fresh frass	T0	10.1 ± 0.2^A,a^	9.9 ± 0.3^A,a^	8.3 ± 0.1^A,c^	8.7 ± 0.0^A,a^
R storage	T0 C1	9.9 ± 0.0^a^	9.0 ± 0.0^bc^	8.7 ± 0.1^a^	8.4 ± 0.0^ab^
	T0 C2	9.3 ± 0.9^a^	9.5 ± 0.2^ab^	8.4 ± 0.1^b^	8.4 ± 0.1^ab^
AT storage	T0 A1	9.4 ± 0.5^a^	8.8 ± 0.3^c^	8.1 ± 0.0^c^	8.3 ± 0.1^ab^
	T0 A2	9.2 ± 0.2^a^	8.4 ± 0.2^c^	7.8 ± 0.0 ^d^	8.0 ± 0.4^b^
Heat‐treated frass	T1	7.2 ± 0.1^B,a^	7.4 ± 0.1^B,a^	5.7 ± 0.4^B,a^	4.9 ± 0.2^B,a^
R storage	T1 C1	7.4 ± 0.2^a^	7.7 ± 0.1^a^	5.7 ± 0.2^a^	4.9 ± 0.2^a^
	T1 C2	7.4 ± 0.1^a^	7.4 ± 0.1^a^	5.6 ± 0.1^a^	4.8 ± 0.4^a^
AT storage	T1 A1	7.4 ± 0.1^a^	7.9 ± 0.2^a^	5.6 ± 0.3^a^	5.3 ± 1.1^a^
	T1 A2	7.6 ± 0.4^a^	7.5 ± 0.1^a^	5.6 ± 0.3^a^	4.7 ± 0.2^a^

		Enterobacteriaceae (log cfu/g)			
Condition	Code	HI5	HI6	TM5	TM6
Fresh frass	T0	7.2 ± 0.2^A,a^	7.1 ± 0.1^A,ab^	6.7 ± 0.1^A,a^	5.9 ± 0.1^A^
R storage	T0 C1	6.6 ± 0.1^b^	7.7 ± 0.3^ab^	6.3 ± 0.2^a^	5.9 ± 0.1
	T0 C2	6.1 ± 0.0^c^	6.4 ± 0.1^b^	5.7 ± 0.1^b^	6.0 ± 0.6
AT storage	T0 A1	< 6.0 ± 0.0^c^	3.2 ± 0.2^c^	5.7 ± 0.2^b^	5.5 ± 0.1
	T0 A2	< 1.1 ± 0.2 ^d^	2.8 ± 0.6^c^	5.5 ± 0.3^b^	6.0 ± 0.6
Heat‐treated frass	T1	< 1.0 ± 0.0^B,a^	< 1.0 ± 0.0^B,a^	2.0 ± 0.5^B,a^	< 1.0 ± 0.0^B,a^
R storage	T1 C1	< 1.0 ± 0.0^a^	< 1.0 ± 0.0^a^	1.1 ± 0.2^b^	< 1.0 ± 0.0^a^
	T1 C2	< 1.0 ± 0.0^a^	< 1.0 ± 0.0^a^	< 1.0 ± 0.0^b^	< 1.0 ± 0.0^a^
AT storage	T1 A1	< 1.0 ± 0.0^a^	< 1.0 ± 0.0^a^	1.8 ± 0.6^ab^	< 1.0 ± 0.0^a^
	T1 A2	< 1.0 ± 0.0^a^	< 1.0 ± 0.0^a^	1.0 ± 0.0^b^	< 1.0 ± 0.0^a^

*Note:* Means of samples for TVC or Enterobacteriaceae counts before (T0) and after heat treatment (T1) within a column, with the same letter (A–B) in superscript do not differ significantly (*p* ≥ 0.05).

Means of samples for TVC or Enterobacteriaceae count with no letter or with the same letter (a–d) in superscript within a column do not differ significantly (*p* ≥ 0.05), for respectively stored fresh (T0, T0C, and T0A) and stored heat‐treated frass (T1, T1C, and T1A).

TVC values for fresh BSFL frass (HI5‐6) were significantly higher than the values for YM frass (TM5‐6), while Enterobacteriaceae counts were comparable. Results were in line with observations in experiments 1 and 2. *Salmonell*a counts in fresh frass samples were below the detection limit of 2.0 log cfu/g and remained unchanged after heat treatment and storage. The previously observed effects of the reference heat treatment were confirmed. TVC reduction was statistically significant (*p* < 0.0001) but residual counts were above 5 log cfu/g. Enterobacteriaceae counts in heat‐treated BSFL frass were below 1.0 log cfu/g. Enterobacteriaceae counts were also significantly reduced in YM frass (*p* < 0.0001), below detection limit for TM6 but less for TM5 (residual count 2.0 ± 0.5 log cfu/g), most likely because of its higher initial load, lower moisture content, and a_w_ value.

Storage of fresh and heat‐treated frass, both refrigerated (4.6°C ± 0.6°C) and at ambient temperature (27.4°C ± 0.8°C), induced less than 1.5 log unit changes in TVC, reduction was statistically significant for some samples, but the microbiological impact was minor. Refrigerated storage of fresh frass resulted in less than 1.2 log unit change in Enterobacteriaceae count. For storage at ambient temperature limited reduction was observed for YM frass, while BSFL frass samples showed significant Enterobacteriaceae count reduction. For HI5 the initial count of 7.2 log cfu/g decreased more than 2 log units after 1 week and to a value below the detection limit after 2 weeks. Reductions for HI6 were 3.9 and 4.3 log units after storage for 1 and 2 weeks, respectively, starting from an initial value of 7.1 log cfu/g. During ambient temperature storage, moisture content for fresh BSFL frass also decreased significantly (*p* < 0.0001), by 5% and 2% for HI5 and HI6, respectively, while for YM frass, this decrease was limited to 1%. A possible explanation for the observed Enterobacteriaceae count reduction in fresh BSFL frass at ambient storage might be outcompetition by other/antagonistic microorganisms resulting from the combined effects of lower water availability and nutrient competition. At lower temperature (refrigerated storage) or lower water activity (YM frass), these species may be unable to proliferate. A second explanation may be found in the observed response to heat treatment in the first experiment. Heating of HI frass above 50°C induces between 3 and 5 log unit reduction in Enterobacteriacea count, while for TM frass, a similar reduction requires a minimum temperature of 70°C. When stored at ambient temperature, fresh BSFL frass, a microbiologically unstable product due to its high moisture content and a_w_ value, will start to compost and heat‐up. Temperatures between 42°C and 58°C may be reached (Deruytter et al. 2022) and induce Enterobacteriaceae count reduction. For YM frass, less prone to spontaneous composting, the achieved temperatures may not be sufficient.

**Figure 4:**
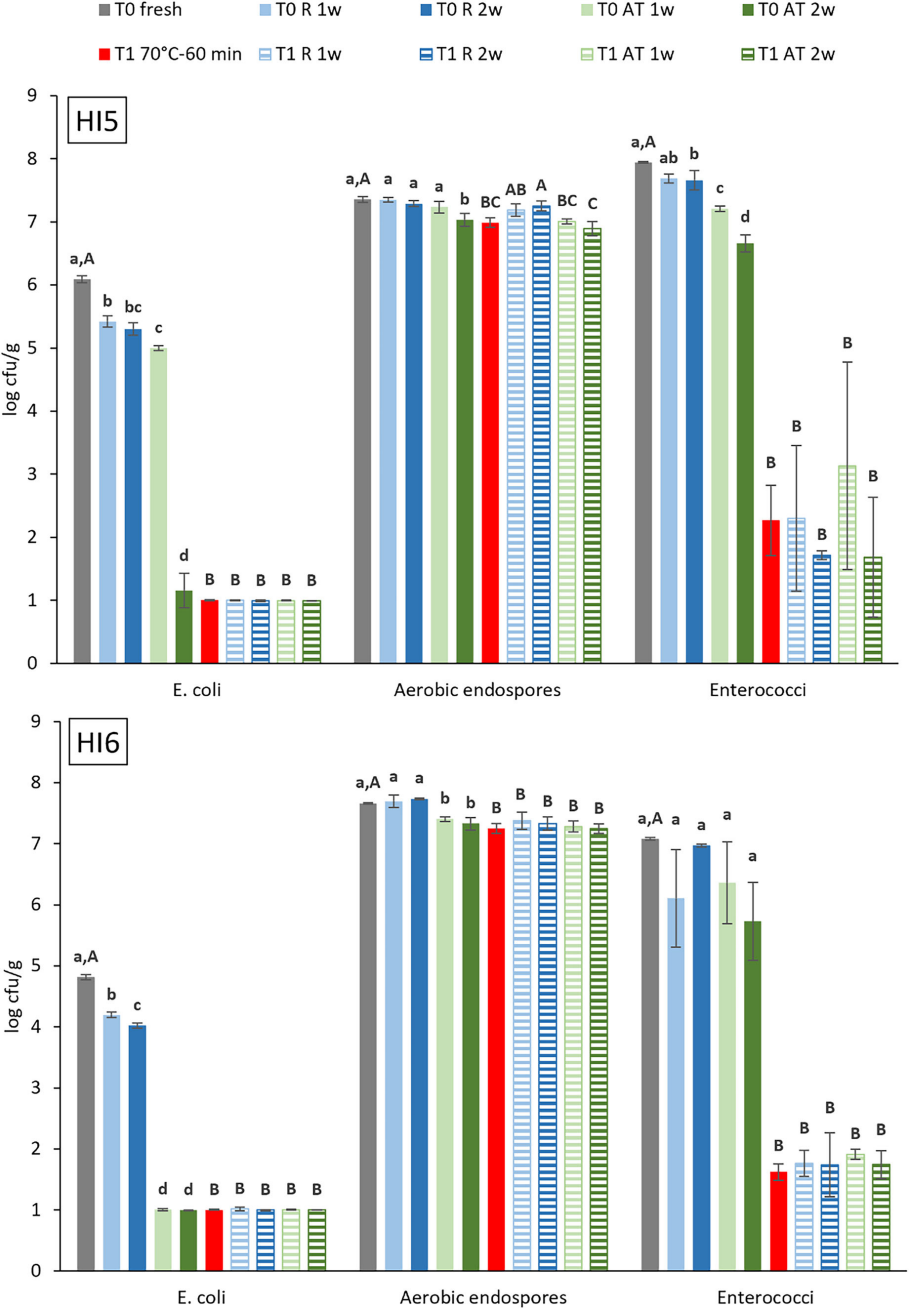
*E. coli*, aerobic endospore and Enterococci counts (log cfu/g) for fresh (T0, gray) and heat‐treated (T1, 70°C–60 min, red) black soldier fly larvae frass HI5 (top) and HI6 (bottom), before (solid fill) and after (striped) storage, refrigerated (R, blue) and at ambient temperature (AT, green), for 1 and 2 weeks. Results represent the mean and standard deviation of 3 replicates. a–d: means of samples (T0 and T0 stored) with the same letter per frass sample do not differ significantly (*p* ≥ 0.05); A–C: means of samples (T0, T1, and T1 stored) with the same letter, per frass sample, do not differ significantly (*p* ≥ 0.05). Detection limit 1.0 log cfu/g. Legal limit for *E. coli* and Enterococcaceae: 3.0 log cfu/g.

*E. coli*, aerobic endospore and Enterococci counts in fresh (T0) and heat‐treated (T1) BSFL frass and their evolution during subsequent storage are illustrated in Figure 4. *E. coli* counts for fresh BSFL frass were 6.1 ± 0.1 and 4.8 ± 0.1 log cfu/g for HI5 and HI6, respectively, and exceeded the legally allowed 3.0 log cfu/g. No comparison data were found for other frass samples, but numbers are lower than or comparable to those reported for cow manure (4.8–7.8 log cfu/g) and chicken litter (6.0–7.4 log cfu/g) (Lin et al. 2022). Since the observed change was less than 0.8 log units, refrigerated storage had a minor influence on *E. coli* count. Storage at ambient temperature induced similar changes as observed for Enterobacteriaceae. *E. coli* counts were significantly reduced by more than 2 log units (HI5) and 3.8 log units (HI6) after 1 week to less than 1.0 log cfu/g after 2 weeks (*p* < 0.0001). Apart from outcompetition and composting heat, the observed reduction of Enterobacteriaceae (including *E. coli*) during ambient temperature storage may be linked to the presence of antimicrobial peptides in the BSFL frass, secreted by the larvae and originating from their gut microbiome. This was also observed in an experiment by Lopes et al. (2020) who inoculated *E. coli* at 6 log cfu/g in BSFL rearing substrate. Reduction of the *E. coli* number was only observed when larvae were present. However, on re‐inoculation of the substrate, the *E. coli* count was reduced even when larvae were no longer present. Pathogen reduction was found to be induced by antimicrobial substances, secreted by the BSFL larvae. Heat treatment for 1 h at 70°C reduced *E. coli* counts below detection limit and subsequent storage did not induce proliferation of the pathogen.

Aerobic endospore counts were 7.4 ± 0.0 and 7.7 ± 0.0 log cfu/g for fresh BSFL samples HI5 and HI6, respectively, and surpass the values of 4.2 to 7.0 log cfu/g reported by Wynants, Frooninckx, Crauwels, et al. (2019) and 5.0 and 5.3 log cfu/g by Van Looveren et al. (2022). Reference heat treatment reduced aerobic endospore count by only 0.4 log units, which was expected as bacterial endospores are generally considered to be heat resistant. Storage of both fresh and heat‐treated frass minimally influenced aerobic endospore number.

Enterococci counts for fresh HI5 and HI6 were 7.9 ± 0.0 and 7.1 ± 0.0 log cfu/g, respectively, which is slightly higher than the average count of 6.0 log cfu/g reported by Swinscoe et al. (2019) for frass from BSFL raised on seaweed powder. Lin et al. (2022) also reported lower Enterococci counts for cow manure (5.8–7.0 log cfu/g) and pig manure (4.3–6.2 log cfu/g). Enterococci count was reduced by 1 log unit during refrigerated storage and by 1.3 log units during storage at ambient temperature, but counts remained well above 3.0 log cfu/g. Heat treatment, however, significantly reduced Enterococci counts in BSFL frass to values below 3.0 log cfu/g. During subsequent storage, values remained below 3.0 log cfu/g except for HI5 where, after 1 week of storage at room temperature, Enterococci count increased to 3.1 log cfu/g but after 2 weeks decreased again to 1.7 log cfu/g. No statistical differences were found between Enterococci counts in the heat‐treated BSFL frass before and after storage. Enterococci, a genus of lactic acid bacteria, are commensals of the gastrointestinal tracts of insects and mammals. The species *E. faecalis* and *E. faecium* are the most prevalent in humans and the latter is often used as a surrogate for *Salmonella* to study thermal inactivation of low water activity foods. Gu et al. (2022) reported 3.7 times higher D‐values for *E. faecium* compared to *Salmonella* in their study on heat inactivation of the pathogens in cornmeal with 16% moisture and 1.5 times higher D‐values at 28% moisture. Based on their D‐value of 0.78 min at 68°C for *E. faecium* in cornmeal at a_w_ 0.75% and 28% moisture, the estimated elimination time of the average Enterococci number of 7.5 log cfu/g for treatment at 70°C of HI5‐6, with an average moisture content of 33%, is only 6 min. Although reduction to counts below 3.0 log cfu/g was observed for the reference treatment, Enterococci were not eliminated, residual average count was 1.9 log cfu/g, contradicting the estimated reduction.

**Figure 5:**
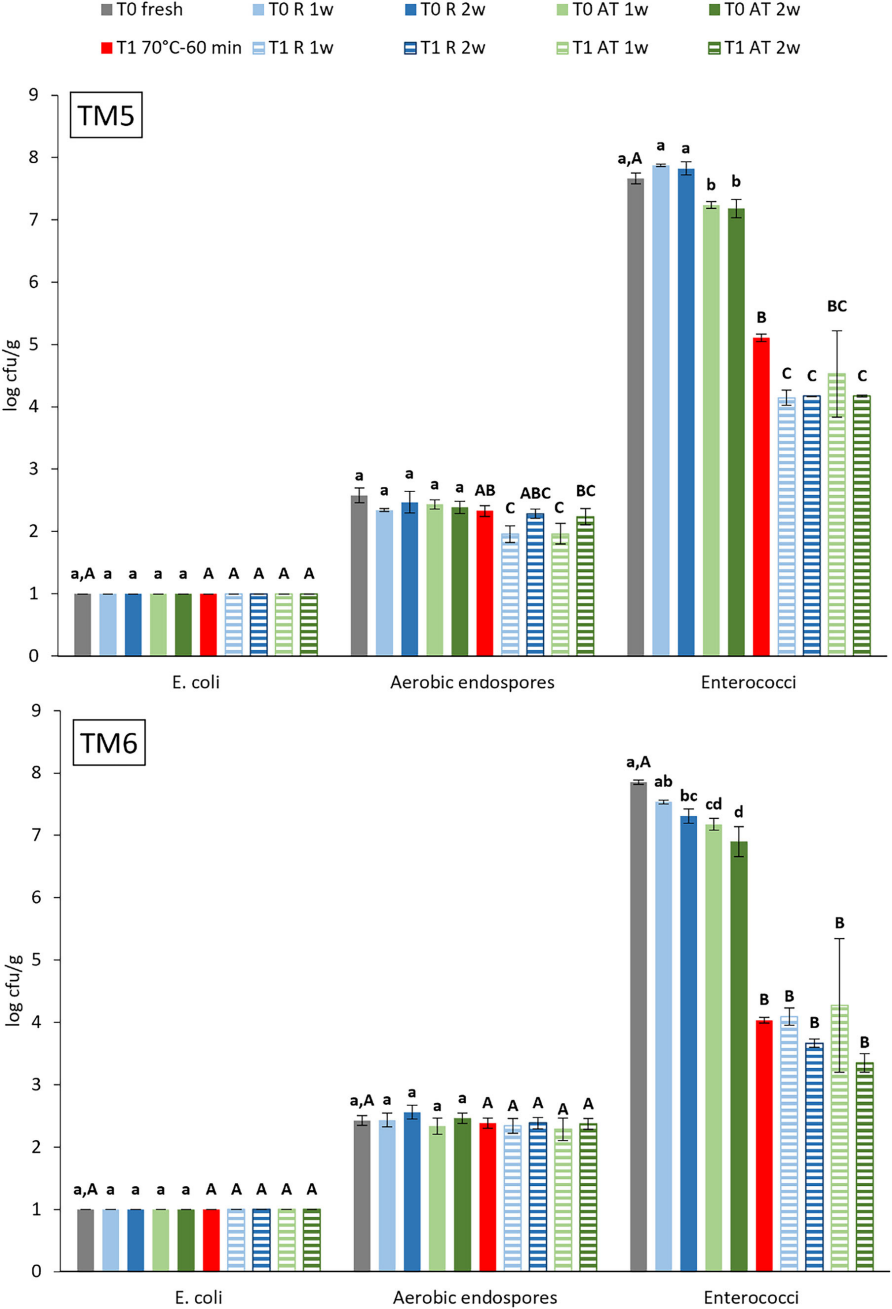
*E. coli*, aerobic endospore and Enterococci counts (log cfu/g) for fresh (T0, gray) and heat‐treated (T1, 70°C–60 min, red) yellow mealworm frass TM5 (top) and TM6 (bottom), before (solid fill) and after (striped) storage, refrigerated (R, blue) and at ambient temperature (AT, green), for 1 and 2 weeks. Data represent mean and standard deviation of 3 replicates. a–d: means of samples (T0 and T0 stored) with the same letter, per frass sample, do not differ significantly (*p* ≥ 0.05); A–C: means of samples (T0, T1, and T1 stored) with the same letter, per frass sample, do not differ significantly (*p* ≥ 0.05). Detection limit 1.0 log cfu/g.

*E. coli*, aerobic endospore and Enterococci counts in fresh (T0) and heat‐treated (T1) YM frass and their evolution during subsequent storage are presented in Figure 5. *E. coli* was not detected in fresh YM frass. Aerobic endospore counts were 2.6 ± 0.1 and 2.4 ± 0.1 log cfu/g in fresh TM5 and TM6, respectively. Endospore counts were significantly lower (4 log units, *p* < 0.0001) than values in fresh BSFL frass and lower than reported values of 3.7 and 5.4 log cfu/g for YM raised on wheatmeal (Osimani et al. 2018). Enterococci counts for fresh YM frass (7.7 ± 0.1 and 7.9 ± 0.0 log cfu/g) were comparable to counts for fresh BSFL frass. The reference heat treatment of YM frass did not significantly reduce aerobic endospore counts. Enterococci count reduction, respectively by 2.6 and 3.8 log units for TM5 and TM6, was significant (*p* < 0.0001). However, reduction was lower than observed for BSFL frass and did not result in counts below 3.0 log cfu/g. Storage of fresh and heat‐treated YM frass did not induce *E. coli* contamination and changed aerobic endospore and Enterococci counts by less than 1 log unit, which was expected given its unfavorable conditions for microbial growth (acidic pH, low a_w_ value, and low‐moisture content).

Overall, the results show that despite the high initial number of all tested micro‐organism in fresh BSFL frass samples, combined with growth permitting near‐neutral pH and a_w_ value of 0.94, storage up to 2 weeks, whether refrigerated or at ambient temperature, did not increase bacteria numbers. Surprisingly, ambient storage had a significant reducing effect on the number of Enterobacteriaceae and *E. coli*. These findings indicate that processing of fresh BSFL frass may be postponed up to 2 weeks. Reference heat‐treated BSFL frass complied with EU criteria to allow use in plant cultivation and may also be stored for at least 2 weeks. For the tested YM frass samples, storage of both fresh and heat‐treated frass up to 2 weeks should also be possible. However, it is important to mention that frass storage in an industrial production setting will differ largely from the experiment conditions, which were aimed at requiring a first insight in the microbial changes during frass storage. Before observations may be generalized, frass samples from different insect species, different production locations, stored in heaps, containers, big bags, or otherwise, should be tested. It will not only be important to assess storage conditions and times for untreated frass, but also to assure that after treatment, no *Salmonella* contamination occurs. EU legislation (Table 2) stipulates that, during or immediately after processing of frass from insect production, the number of *E. coli* or Enterococcaceae should be below 3.0 log cfu/g. Our findings indicate a different response to heat treatment for frass from the production of both insect species. While for *E. coli*, when present in fresh BSFL or YM frass, counts could be sufficiently reduced, this did not apply to Enterococcaceae, often present in larger numbers. However, to allow use in plant cultivation, the left‐over substrate from insect rearing must only comply with either one of both criteria.

## 4 Conclusions

This study investigated the microbiological quality and safety of frass, originating from the production of BSFL and YMs, aimed for use as fertilizer and/or soil amendment in crop production. Fresh BSFL and YM frass have high Enterobacteriaceae counts but show no *Salmonella* contamination. *E. coli* numbers in BSFL frass are higher compared to YM frass, while Enterococci counts are similar and above 3.0 log cfu/g. Untreated BSFL frass did not comply with the microbiological safety standards, some YM frass samples did. After reference heat treatment, frass from the rearing of both insect species was found safe for fertilizer use. In BSFL and YM frass, *E. coli* counts were reduced below the detection limit. Reduction of Enterococci counts below 3.0 log cfu/g was only achieved for BSFL frass. However, to allow use in plant cultivation, insect frass must only comply with either the *E. coli* or the Enterococcaceae criterion. Considering milder treatments, BSFL frass complied with the criteria if a minimum treatment temperature of 60°C was applied. For YM frass, treatment at temperatures below 70°C insufficiently reduced Enterobacteriaceae and (inoculated) *Salmonella*. Fresh and reference heat‐treated frass from both insect species could be stored up to 2 weeks, in refrigerated conditions (4°C) and at ambient temperature (26°C–28°C), without increasing bacterial counts.

Even though current EU legislation does not differentiate frass samples from the production of separate species, obtained results indicate variations in intrinsic characteristics and microbial load, as well as in response to heat treatment and storage, for frass from BSFL and YM rearing. Furthermore, similar conclusions can be drawn for frass from the same species, reared on different substrates, originating from diverse production facilities, with a distinct matrix composition, carrying a different microbial load. This emphasizes the necessity for development of “tailor‐made” treatments for insect frass, which, based on the actual microbial load and properties, ensure microbiological safety whilst preserving fertilizer and biostimulant potential. This would benefit the insect‐producing sector. Avoiding unnecessary or excessive, energy‐consuming heat treatments of frass would reduce production costs and generate a higher quality, more competitively marketable fertilizer. YM frass, low in *E. coli* numbers and exhibiting microbial growth restricting properties, will most likely meet the microbiological safety criteria without treatment. When properly stored, preventing *Salmonella* contamination, it may be marketed without additional costs for insect producers. BSFL frass, on the other hand, because of its higher microbial load and instability, will require treatment, not only to ensure microbiological safety but also to reduce moisture content and make it more suitable for handling and application in agriculture. This may well be a heat treatment, possibly at lower temperatures or for a shorter time. Exploring other options, such as heap or reactor composting or a combination of techniques, may be advantageous. Given the observed low contamination with actual food pathogens, establishing a more specific microbiological risk profile for frass from insect production may be appropriate. Less strict regulations may again lower the need for treatment.

EU legislation does not differentiate between “farmed” insects and traditionally farmed livestock, thereby limiting the range of allowed feeding substrates. An expansion to the use of post‐consumer food waste would lower input costs, secure input supply, and improve the sectors circularity by reintroducing more “food waste” into the food chain. Although these types of rearing substrates may potentially carry a higher pathogen load, it should be possible to ensure microbiological safety of the residual frass by developing customized treatment methods.

## Author Contributions

**Ann De Volder:** conceptualization (lead), writing – original draft (lead), formal analysis (lead), writing – review and editing (equal). **Jeroen De Smet:** writing – review and editing (equal). **Lotte Frooninckx:** writing – review and editing (equal). **David Deruytter:** writing – review and editing (equal). **Johan Ceusters:** writing – review and editing (equal). **Dries Vandeweyer:** conceptualization (lead), writing – original draft (supporting), formal analysis (supporting), writing – review and editing (equal).

## Ethics Statement

The authors have nothing to report.

## Conflicts of Interest

The authors declare no conflicts of interest.

## Data Availability

The data that support the findings of this study are openly available in Lirias at 10.48804/LO6MOZ. Data supporting this study are openly available from Lirias (Data Repository of KU Leuven University) at 10.48804/LO6MOZ.
